# Quality Management Systems Implementation Compared With Organizational Maturity in Hospital

**DOI:** 10.5539/gjhs.v8n3p174

**Published:** 2015-07-27

**Authors:** Tayebeh Moradi, Mehdi Jafari, Mohammad Reza Maleki, Seyran Naghdi, Hesam Ghiyasvand

**Affiliations:** 1 Research Center for Health Services Management, Institute for Futures Studies in Health, Kerman University of Medical Sciences, Kerman, IR Iran; 2 School of Health Management and Information Sciences, Iran University of Medical Sciences, Tehran, IR Iran; 3 Research Center for Social Determinants of Health, Institute for Futures Studies in Health, Kerman University of Medical Sciences, Kerman, Iran; 4 Research Center for Modeling in Health, Institute for Futures Studies in Health, Kerman University of Medical Sciences, Kerman, Iran

**Keywords:** organizational maturity, Quality Management System, ISO 10014, hospital

## Abstract

**Background:**

A quality management system can provide a framework for continuous improvement in order to increase the probability of customers and other stakeholders’ satisfaction. The test maturity model helps organizations to assess the degree of maturity in implementing effective and sustained quality management systems; plan based on the current realities of the organization and prioritize their improvement programs.

**Objectives:**

We aim to investigate and compare the level of organizational maturity in hospitals with the status of quality management systems implementation.

**Materials and Methods:**

This analytical cross sectional study was conducted among hospital administrators and quality experts working in hospitals with over 200 beds located in Tehran. In the first step, 32 hospitals were selected and then 96 employees working in the selected hospitals were studied. The data were gathered using the implementation checklist of quality management systems and the organization maturity questionnaire derived from ISO 10014. The content validity was calculated using Lawshe method and the reliability was estimated using test - retest method and calculation of Cronbach's alpha coefficient. The descriptive and inferential statistics were used to analyze the data using SPSS 18 software.

**Results:**

According to the table, the mean score of organizational maturity among hospitals in the first stage of quality management systems implementation was equal to those in the third stage and hypothesis was rejected (p-value = 0.093). In general, there is no significant difference in the organizational maturity between the first and third level hospitals (in terms of implementation of quality management systems).

**Conclusions:**

Overall, the findings of the study show that there is no significant difference in the organizational maturity between the hospitals in different levels of the quality management systems implementation and in fact, the maturity of the organizations cannot be attributed to the implementation of such systems. As a result, hospitals should make changes in the quantity and quality of quality management systems in an effort to increase organizational maturity, whereby they improve the hospital efficiency and productivity.

## 1. Background

Nowadays, the quality and its promotion in hospitals is one of the important issues in health system management; so that the quality assurance of health services is considered as a part of governments’ mission, one of the important tasks of health managers in countries as well as one of the main priorities in health sector reforms (Øvretveit, 2001; Marshall et al., 2006). In addition, the quality of services in hospitals as the most important element of the health system has a special status (McKee & Healy, 2000). Quality management system is a management system to direct and control an organization with regard to quality issue (Jing, 2010). There are many definitions for such a system; a quality management system in itself is not able to make the organization more profitable, efficient and customer-oriented, but rather enables the organization to perform better than before (Aggelogiannopoulos, Drosinos, & Athanasopoulos, 2007). In other words, the quality management system can be viewed as a complex system of all parts and components of an organization that focuses on quality processes and activities (Lombarts, Rupp, Vallejo, Klazinga, & Suñol, 2009).

Currently, organizations need to have a new management tools and approaches in order to reconcile traditional evaluation methods with new and more prospective management methods. Although hospitals are one of the most complex industries, for now are managed using traditional methods (Van den Heuvel, Koning, Bogers, Berg, & van Dijen, 2005). The quality management systems approach encourages organizations to consider customer requirements, determine processes affecting the fulfillment of product or service accepted by customers and controls these processes (Lee, To, & Yu. 2009).

In recent years, many efforts have been made to improve the quality of health services in the country. Previous experiences suggest that although these programs have had undeniable positive effects, they have typically been involved in fragmentation and discontinuity in quality improvement processes due to lack of systematic and holistic approach. ISO standardization methods, implementation of total quality management programs, establishment of complaints-handling offices, client reference programs, excellence models such as EFQM and recently, clinical governance system are among plans with different approaches which have been announced and implemented with the aim of improving healthcare organizations and their services levels (Kaplan et al., 2010).

An appropriate quality management system is a structural framework which every organization should establish it prior to delivery products or provide services to their customers or consumers (Linderman, Schroeder, Zaheer, Liedtke, & Choo, 2004). Although common methods and concepts of quality management and excellence, such as ISO, 5S, EFQM and so on are useful, their ability to have a profoundly impact on the health sector should be questioned (Kaplan et al., 2010; Buciuniene, Malciankina, Lydeka, & Kazlauskaite, 2006).

S et al. in 2007, in a study titled “how Hospitals Choose a Quality Management System: Relevant Criteria in Large Spanish Hospitals” examined the implementation of three quality management system models including ISO 9001, Joint Commission (JC), EFQM, or a combination of them and selection criteria affecting Spanish hospitals of size > 400 beds when choosing a quality management system. According to the study, the term implementation of quality management systems refers to running the ISO 9001 model, hospital accreditation system and European model for Organizational Excellence (EFQM) or a combination of them. To gather the required data, the researchers sent a questionnaire to 101 hospitals. The results of the study showed that in the studied hospitals, 71.4% has used ISO 9001 quality management system; 11.9% JC, and 69% EFQM (Sangüesa, Mateo, & Ilzarbe, 2007).

Furthermore, organizations use models of organizational maturity to know whether they will continue their route towards maturity. Maturity models provide reference points for the organization to evaluate itself (or for external evaluation) based on best practices according to one or more specific guidelines (Nazar & Abbasi, 2009; Smith, 2010). The test maturity model helps organizations to assess the degree of maturity in implementing effective and sustained quality management systems; plan based on the current realities of the organization and prioritize their improvement programs (Eileen et al., 2005). Also, this model will help organizations to identify prerequisites for implementing the quality management system according to the maturity of the organization (Cooke-Davies & Arzymanow, 2003). Some of the maturity models provide limited guidance on how to achieve more maturity and most of them are in such a way that the users themselves must find the path to higher levels of maturity. Some of these models include capability Maturity Model Integration, organizational project management maturity model, ISO 9004.

One of them is self-assessment can provide a general overview of the organization performance and the degree of maturity of its management system. The self-assessment also can help organizations to identify areas needing improvement or innovation and prioritize next initiatives (ISO 10014, 2006; Wolniak, 2013; Boys, Karapetrovic, & Wilcock, 2004). The results of the assessment can be used as input to management investigations (ISO 9004, 2009). In this model, derived from the standard 10014, an organization is evaluated in eight dimensions to assess its maturity and readiness to accept the effective and efficient organization's quality management system. The dimensions are as followed: customer focus, leadership, employee involvement, process approach, system approach to management, continuous improvement, realistic approach to decision-making and communication with suppliers based on mutual interests.

There is a combination of 8 principles of the quality management, the plan–do–check–act cycle (PDCA) and process approach in this standard. Self assessment according to ISO 10014 considers five levels of maturity (level 1 represents the lowest level of maturity while level 5 is the highest). In this model, the maturity of an organization's quality management is reflected based on the presence or absence of balanced or unbalanced development of the abovementioned dimensions (ISO 10014, 2006).

**Diagram 1:**
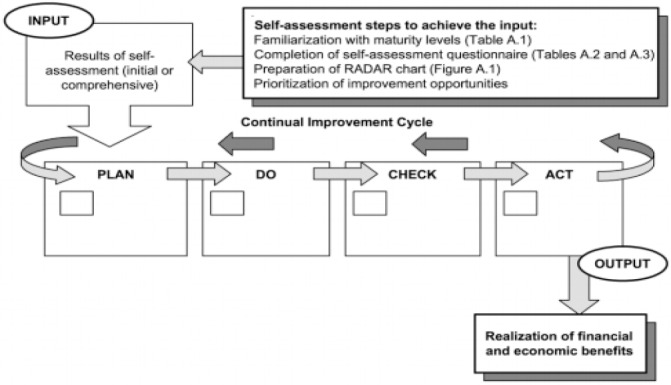
Generic representation of the overall process in ISO 10014

In fact, this study may be a response to a question about why some efforts made in the field of implementing quality management systems in some organizations have not expectedly succeeded and the quality management systems in these organizations are survived just symbolically and apparently with the efforts and support by only a few individuals while in some organizations, the opposite happens.

According to the studies on this area of research, there has been no study on the relationship between the two variables, organizational maturity and implementation of quality management systems, in hospitals, both inside and outside the country yet. Most studies have examined only one of these two variables. While studies in this area have been conducted mainly in non-medical service organizations especially in the IT field, this study has examined the importance of organizational maturity dimensions and quality management systems in the hospital as the main healthcare services provider in addition to provide new insight into them.

## 2. Objectives

This study has investigated and compared the status of quality management systems implementation with the level of organizational maturity in selected hospitals in Tehran using the eight quality management principles.

## 3. Materials and Methods

This descriptive-analytical cross-sectional study considered from applied perspective has employed qualitative methods and been conducted in hospitals with over 200 beds located in Tehran. This study was carried out in two methodologically independent steps; accordingly, all elements of the study have been described separately for each step.

### 3.1 Step One: Determining the Status of the Quality Management Systems Implementation

The first setting of the study has been all hospitals in Tehran. Given that in practice, small hospitals do not follow such quality management system (Sangüesa, Mateo, & Ilzarbe, 2007) and there are a large number of hospitals in Tehran, the inclusion criteria for entering hospitals into study were determined as below:

1. Being located in Tehran

2. Having 200 beds and above.

There have been a total of 39 hospitals but seven hospitals of them were not willing to participate in the study. In terms of ownership, there were 21 public, 4 private, 4 Social Security Organization hospitals as well as 4 hospitals owned by other organizations.

At this step, in order to collect data about the implementation of quality management systems in studied hospitals, a checklist to investigate the current status of hospital has been used; This checklist included questions about the hospitals’ characteristics and implementation steps of different quality management models in the studied hospitals such as: series of ISO standards (9001, 14001, and 18001), FOCUS-PDCA, EFQM and so on. The main objective of this step of the study is to classify hospitals based on quality management systems. Based on implementation steps of each of quality management models, we get the mark of each of models, from 2 to 6 that it depends on them. Then according to total score, we divide them into three groups. Therefore, hospitals with the points less than 11.7 were classified into level one, between 11.7 to 17.3, level tow and higher than 17.3, level three. Accordingly, in terms of the implementation of the quality management systems, 18 hospitals classified into the first and third levels were entered the second step of the study i.e. the completion of organizational maturity questionnaire.

### 3.2 Step Two: Measuring Organizational Maturity

At this step, all administrators and quality experts working in the first and third levels hospitals located in Tehran have been considered. Due to the limited number of them, only one inclusion criterion was used which was selecting those with one or more years job experience in the hospital since they should be relatively familiar with the hospital and its environment. Accordingly, a total of 96 hospital administrators and quality experts (including experts working in offices of the quality improvement, clinical governance, R&D, hospital experts) were selected in this step. Totally, average of 5 persons per hospital was questioned. The exclusion criterion of the study subjects was non-completion of the questionnaire after three times follow-up during 15 days and the return rate for the questionnaires in this study was 86.46 percent. In order to assess the level of organizational maturity, the standard 10014 designed questionnaire including the eight quality management principles (customer focus, leadership, employee involvement, process approach, system approach to management, continuous improvement, realistic approach to decision-making and communication with suppliers based on mutual interests) was used and the organizational maturity from the hospital administrators and quality experts’ viewpoints on a five-level Likert scale (very high = 5, and very low = 1) was assessed.

Each question in the questionnaire (including 76 questions) was questioned with three options “relevant and important”, “can be used but is not necessary” and “irrelevant”. After receiving the forms, the content validity ratio (CVR) and its index for the questionnaire were calculated using Lawshe method (Lawshe, 1975; Wynd, Schmidt, & Schaefer, 2003). Lawshe model is a way of achieving content validity and has been used in different fields such as health care. It involves a panel of subject matter experts considering the importance of individual items within an

instrument (Ayre & Scally, 2014). After calculating the CVR, 12 questions with CVR less than 0.8 were removed. Then, the questionnaire was distributed among experts again and according to the experts’ opinion, 10 questions were merged and 13 questions with the CVR less than 0.8 were removed. Finally, the questionnaire was finalized with 41 questions.

To assess the reliability using the test- retest method, the questionnaire was distributed among 10 hospital administrators and quality improvement experts working in the selected hospitals and the questionnaire, once again, was distributed among the same individuals after two weeks; the Cronbach's alpha coefficient was equal to 0.843. Given that the closer Cronbach's alpha coefficient is to 1.0 the greater the internal consistency of the items in the scale; this value indicates the strong reliability due to high internal consistency of the questions in the questionnaire. To complete the organizational maturity questionnaire, the researcher visited to all studied hospitals in the first step of the study in person and after providing the introduction letter, distributed the questionnaire among the selected hospital administrators and experts in quality and clinical governance while describing the nature of the project and ensuring the confidentiality of their responses to get consent to participate. To facilitate responding, the research information form was also attached to the questionnaire in order to have access to the researcher and get answers to questions in the case of any question about the study and the questions. In this study, the implementation of quality management systems and the organizational maturity have been considered as the dependent variable and the independent variable, respectively. To analyze the collected data, in the first step, the output data about the implementation of quality management systems was obtained in three features. In the second step, the data about the organizational maturity level based on eight quality management principles were gathered on a five-point Likert scale. In the third step, the relationship between the organizational maturity and the implementation of quality management systems in studied hospitals was examined.

The descriptive statistics (frequency ratio - the percentage, mean, variance and standard deviation) in the form of statistical tables, graphs and numerical indicators were used in order to analyze the data using the statistical software SPSS-18 and Excel. In order to assess the organizational maturity level and implementation of quality systems, first, the normality distribution of the response variables was examined using the Kolmogorov-Smirnov test. Then, the T-test and Mann-Whitney U test were used for the inferential analysis of the relationship between various dimensions of the organizational maturity and the first and third level hospitals in the implementation of quality management systems. Using the mean value can neutralize the effect of unequal sample size. If the p-value is less than 0.05, there is a significant difference between the eight dimensions of the organizational maturity in the first-level and third-level hospitals in implementing the quality management systems.

## 4. Results

In this study, all the systems implementing successfully, involving the establishment, having certification or documentation indicating successful implementation of the quality management models have been considered as the variable for the implementation of quality management systems. As shown in Table 1, the accreditation model with a maximum value of 78.13% (n = 25) and ISO 14001 with a minimum value of 3.13% (n = 1), respectively, have been the highest and the lowest quality management systems used in the studied hospitals.

**Table 1:** The distribution implementation of the quality management systems in studied hospitals

Quality Management Systems(QMSs)	Frequency	Percentage
**Accreditation**	25	78.13
**ISO 9001**	17	53.13
**EFQM**	13	40.63
**BSC**	6	18.75
**5S**	5	15.63
**FOCUS-PDCA**	5	15.63
**ISO 18001**	2	6.25
**ISO 14001**	1	3.13

Aside from the accreditation, the two models of ISO 9001 with value of 53.13% (n= 17) and the European model for Organizational Excellence (EFQM) with value of 41.63% (n=13) have been identified as the most frequently used models in the hospitals. The remaining models have been implemented or are being implemented in less than 20 percent of the hospitals.

The status of studied hospitals in terms of the implementation of the quality management system has been illustrated in Table 2.

**Table 2:** The levels of implementation of the quality management system in studied hospitals

Level of Hospital	Range of Score	Frequency	Percentage
**First Level**	11/7	14	43.75
**Second Level**	11/7-17/3	14	43.75
**Third Level**	17/3>	4	12.50
**Total**	-	32	100.00

In the second step of this study, 83 subjects participated. According to the figure illustrating the scores of the organizational maturity in the hospitals and their level of the quality management systems implementation, the dimensions of the customer focus and the leadership (3.54) have had the highest average while the dimension of the system approach to management has had the lowest average (3.18). The findings of the study showed that the mean score of the eight dimensions of the organizational maturity among hospitals in the third level hospitals has been higher than that in the first level hospitals. Table 3 shows the information about the frequency and the percentage frequency of the three different levels of the organizational maturity (low, medium and high) in the hospitals at the first and third level of implementing the quality management systems as well as all hospitals. Given that the scores of the organizational maturity in hospital have been between1.10 to 4.51, the scores less than 2.5, 2.5-3.5 and more than 3.5 were considered as the low, medium and high organizational maturity, respectively. According to the table, while the third-level hospitals has not had low organizational maturity and almost 55 percent of high organizational maturity has been attributed to them, the first-level hospitals have had 36.5% of the high organizational maturity.

**Table 3:** The three levels of the organizational maturity in the first and third - level hospitals at implementing the QMSs

Organizational Maturity	The first-level hospitals		The third-level hospitals		Total of Hospitals	
		
	n	%	n	%	n	%
**Low**	3	5.8	0	0	3	3.6
**Medium**	30	57.7	14	45.2	44	53
**High**	19	36.5	17	54.8	36	43.4
**Total**	52	100	31	100	83	100

The dimensions of the organizational maturity in the first- and third-level hospitals have been displayed in the following RADAR chart. According to the chart, the mean score for the eight dimensions of organizational maturity in third-level hospitals has been higher than that in the first level hospitals. The advantage of the RADAR chart compared to other charts is that the comparison of various positions of the chart at one level is possible.

The mean and standard deviation of the variable of the organizational maturity in terms of the levels of the quality management system implementation and the p-value of the test have been shown in Table 4.

**Diagram 2:**
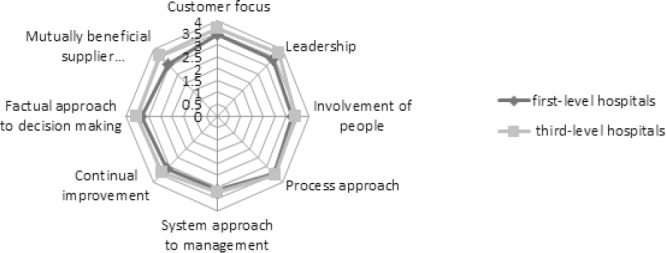
The dimensions of organization in first-level hospitals and in third-level hospitals

**Table 4:** Compare quantity of the organizational maturity in terms of the levels of the quality management system implementation in studied hospitals

Organizational Maturity	Mean	SD
**First-level hospitals**	3.29	0.08
**Third- level hospitals**	3.53	0.10
**P-value(Confidence Interval)**	0.093(-0.515-0.040)		

As it has been illustrated in Table 4, the mean (SD) of the organizational maturity in the first-level hospitals and the third- level hospitals have been equal to 3.29 (0.08) and 3.53 (0.10), respectively.

## 5. Discussions

The findings of the study suggest that the most commonly used models among the studied system included accreditation model (has been announced by the Ministry of Health and Medical Education), ISO 9001, European model for Organizational Excellence and Balanced Scorecard, respectively. Meanwhile, the two models of ISO 14001 and OHSAS have been rarely used. The findings of this study are in consistence with those of Hashemzehy's study on the use of systems or approaches to evaluate the performance (i.e. quality management systems) in 2010. The results of that study showed that among the studied systems, two models of the organizational excellence and the Clinical Governance (43.8%) have been the most widely used systems and the implementation of the ISO approaches and the Balance Scorecard method (25% and 18.8%, respectively) has also been used considerably. The use of the Balanced Scorecard method by as much as 18.8% in Tehran hospitals seems to be quite interesting and beyond the expected (Hashemzehi, Iran nezhadparizi, & Tabibi, 2010); this value is close to the finding of the current study (18.75%). The results of a study titled “how Hospitals Choose a Quality Management System” by Sangüesa et al. in 2007, have been also similar to the results of the current study; so that the ISO (71.4%) has been identified as the most commonly used model and aside from ISO, the EFQM (69%) has been considered as the second widely used quality management system in Spain. Currently, the efficiency of the models of the quality management system has been completely approved; since the implementation of these models in many organizations have led to surprising results and has become a competitive advantage. But still the lack of effectiveness of the implementation of the mentioned models is questioned (Cooke-Davies & Arzymanow. 2003).

The results of this study also indicate that the scores on all dimensions of the organizational maturity are in the range between 3 and 4. In other words, the organizational maturity level in the studied hospitals can be regarded as average to good. However, at the first look, it seems that there should be a significant difference between the organizational maturity in different levels of implementing the quality management systems, according to the equality between the average organizational maturity between the first-level hospitals and the third-level ones in implementing the quality management systems (despite the mentioned default hypothesis) the hypothesis is rejected (p-value = 0.09). According to the findings of a study by Sower et al. in 2007, there is a significant relationship between the quality system maturity and the cost of quality usage by organizations. This study also suggested that the total cost of quality (COQ) declines as an organization's quality system matures. Thus the assessment of the organizational maturity in hospitals and health centers can be considered important and necessary (Sower, Quarles, & Broussard, 2007).

Observations and studies comparing hospital industry with other service industries have shown that health care providers will take a longer journey on the path towards quality improvement in order to achieve positive financial performance. Studies show that most of the major hospital systems which are not well financed are now in the phase of the intermediate stages of the quality evolution (maturity). Small hospitals (with simple facilities) are usually not well financed and are generally in the early stages. On the other hand, a substantial percentage of innovative healthcare systems (creative) are in the middle of the third phase of the quality maturity. Not surprisingly, a very few hospitals are near the final stages (Performance Strategy Change Improvement [PSCI], 2010).

Among the eight dimensions of organizational maturity, only the three ones including “customer focus”, “leadership” and “communication with suppliers based on mutual interests” were significantly different between the first-level and third-level hospitals of implement quality management systems; but the value is also not so high. In other words, implementation of the studied quality management systems has impact on only three dimensions out of the eight organizational maturity dimensions and has affected these three dimensions more than others.

Establishment of quality management models in hospitals is essential for their excellence; but the philosophy and purpose of its establishment in the organization should always be considered. On the other hand, in the case of lack of support from top management for implementation of quality approaches in organizations, not only the customer satisfaction does not improve but also costs and a waste of time is imposed on organizations. Maturity models can help managers design an appropriate quality management system in order to achieve successes in the current situation and also help hospital officials to prioritize corrective actions and develop improvement strategies. As a result, hospitals should make changes in the quantity and quality of quality management systems in an effort to increase organizational maturity, whereby they improve the hospital efficiency and productivity. In fact, the organization maturity can be considered as the same as the organization effectiveness in the establishment and implementation of quality management systems in the organization.
